# Prevalence of Radiolucent and Radiopaque Jawbone Lesions Using Panoramic Radiographic Analysis in Hail, Saudi Arabia: A Retrospective, Cross-Sectional, Observational Study

**DOI:** 10.7759/cureus.69533

**Published:** 2024-09-16

**Authors:** Ibtihag S Elnaem, Yosef Alanazi, Albandri M Alghris, Layla H Alenzi, Ghadah D Aldkhayel

**Affiliations:** 1 Oral and Maxillofacial Surgery, College of Dentistry, University of Hail, Hail, SAU; 2 Dentistry, College of Dentistry, University of Hail, Hail, SAU

**Keywords:** hail, jawbone lesions, panoramic radiography, prevalence study, radiolucent lesions, radiopaque lesions, saudi arabia

## Abstract

Background

Oral diseases are among the most prevalent public health issues worldwide, underscoring the importance of early diagnosis and effective prevention programs. Determining the prevalence of jawbone lesions is crucial for developing targeted interventions and ensuring timely treatment. Panoramic radiography, also known as orthopantomogram, has become a cornerstone in radiographic examinations, offering a comprehensive view of the dental and maxillofacial regions. Its accessibility and ability to detect a wide range of pathologies make it an invaluable diagnostic tool. This study aimed to assess the prevalence and distribution of radiolucent and radiopaque jawbone lesions in the population of Hail, Saudi Arabia, using panoramic radiographic analysis.

Methodology

A retrospective, cross-sectional study was conducted using pre-existing panoramic radiographs of individuals aged 18 years and older in Hail, Saudi Arabia. The radiographs were analyzed for the presence of jawbone lesions, classified as radiolucent, radiopaque, or mixed, and their anatomical distribution was recorded. Statistical analysis was performed using the chi-square test, with a significance level set at p-values <0.05.

Results

A total of 389 jawbone lesions were identified in 177 subjects, representing a prevalence of 45.5%. Radiolucent lesions were the most common, observed in 153 (39.3%) subjects, while radiopaque lesions were found in 18 (4.6%) subjects, and mixed lesions in six (1.5%) subjects. The mandible was more frequently affected than the maxilla, with 104 (59%) lesions occurring in the mandible and 52 (29%) lesions in the maxilla. The posterior region of the jaw was the most commonly involved site, accounting for 124 (31.9%) cases. Gender and age did not significantly influence the prevalence or type of lesions, although a higher incidence was noted in the 31-45-year age group.

Conclusions

This study revealed a significant prevalence of jawbone lesions in the Hail population, with radiolucent lesions being the most common type, predominantly affecting the posterior region of the mandible. The findings highlight the importance of early detection and targeted dental health initiatives in this region. Further research, particularly longitudinal studies, is recommended to explore the natural history of these lesions and their impact on oral health over time.

## Introduction

Jawbones are considered unique compared to other bones in the body. Their development originates from the embryonic neuroectoderm, and, anatomically, they hold the tooth germs. Due to these unique developmental characteristics, diseases in the jaws occur differently from those in other skeletal bones. Therefore, as part of their specialty, dentists should pay close attention to lesions within the maxillofacial region [1]. Lesions in the maxillofacial region encompass a range of neoplastic and non-neoplastic conditions when considering disorders affecting the jawbone [2]. Oral diseases are widely regarded as one of the most prevalent public health issues worldwide, carrying significant socioeconomic consequences [1]. This highlights the need for the diagnosis of these lesions, which is achieved through a sequence of steps, starting with history taking and continuing through to relevant investigations.

As one of the important investigations in jawbone lesions, radiographic examination provides essential information describing their margins, shapes, locations, and associations with adjacent structures [3]. Panoramic radiography, also known as orthopantomogram (OPG), is considered the main element in radiographic examination nowadays, as it allows screening of all teeth, the temporomandibular joint, and both the maxillary and mandibular jaws [4]. Panoramic radiography has the disadvantage that it cannot identify bone invasion until the mineral content has decreased by 30% [5]. However, panoramic radiography provides numerous advantages, including fast acquisition time and the convenience of inspection. Furthermore, its ability to provide comprehensive information about the oral and maxillofacial regions makes it a first-line dental screening tool [6,7]. Additionally, panoramic radiographs are capable of determining and comparing the measurements of the right and left sides of the upper and lower jaws [8]. Although most jawbone lesions are found accidentally during routine patient radiographic examinations, identification of these lesions is essential for all dentists, as diagnosis and treatment are among their responsibilities [9]. Despite its limitations, panoramic radiography remains a widely used diagnostic tool due to its accessibility and ability to provide a comprehensive overview of the dental and maxillofacial regions. It plays a crucial role in the early detection of various pathologies, including both radiolucent and radiopaque lesions, thereby facilitating timely intervention and management.

Determining the prevalence of jawbone lesions is essential for implementing prevention programs and ensuring early diagnosis and treatment, as well as aiding in limiting the progression of conditions that might otherwise complicate a patient’s health. A previous study identified the prevalence of jawbone lesions in the Bagalkot population at 30.62% in 2017 [10]. As unique regional factors such as geographic, genetic, environmental, and lifestyle influences can contribute to variations in disease prevalence and presentation, identifying the prevalence in this region is crucial. To our knowledge, no study has identified the prevalence of jawbone radiolucent and radiopaque lesions in Hail, Saudi Arabia. Therefore, this study aims to measure the prevalence of these lesions in Hail, Saudi Arabia, through panoramic radiographic analysis.

## Materials and methods

This retrospective, observational, cross-sectional study was conducted on a sample from Hail, Saudi Arabia. The study aimed to determine the prevalence of jawbone radiolucent and radiopaque lesions in Hail, Saudi Arabia, using panoramic radiographic analysis achieved through the following objectives: determining the age and gender more susceptible to bone lesions, identifying the jaw affected more by lesions, and determining the predictable site for lesions within the jaws. The inclusion criteria included clear, non-distorted panoramic images of individuals aged 18 years and older who had no syndrome or disease affecting the jawbone and were residents of Hail, Saudi Arabia. Patient records fulfilling the inclusion criteria were selected and those not meeting the inclusion criteria were excluded. The sample was chosen by reviewing files stored in the clinical software system during the period 2022-2024, with random selection conducted by the co-authors. The software system used for sample selection and image analysis was Sirona Dental System GmbH (SIDEXIS XG, version 2.63), operating on a Windows platform. Panoramic images, age, and gender were extracted from these files. Panoramic images were analyzed using the previously mentioned system to complete a preformed checklist. The checklist included gender, age, and the presence or absence of jawbone lesions. If jawbone lesions were present the following information was extracted: radiolucent/radiopaque or mixed, upper/lower or both jaws, right/left or both sides, anterior/posterior or both locations, and teeth associated with the lesion. The sample size was determined to include 386 images at least according to a previous study. To ensure validity between the investigators under the guidance of the supervisor, co-authors randomly analyzed 10% of the sample (40 panoramic images). Afterward, the principal investigator reviewed these images. The results were within the acceptable range, and researchers proceeded to evaluate the remaining images.

Ethical considerations

This study was ethically approved by the Institutional Review Board at the University of Hail (approval number: H-2024-400). As this study utilized previously obtained panoramic radiograph images and did not involve direct interaction with human participants, informed consent was not required. The images analyzed for this research were collected as part of routine clinical procedures and were de-identified to ensure patient privacy and confidentiality. No new data collection or patient interaction occurred during the study. The use of pre-existing images for research purposes falls within the scope of standard practice for secondary data analysis, provided that patient identities remain protected and that the study adheres to ethical guidelines for the use of medical records.

Statistical analysis

The data were gathered, organized, and analyzed using SPSS software for Windows version 21.0 (IBM Corp., Armonk, NY, USA). For data analysis, the chi-square test was used, and a p-value <0.05 indicated statistical significance.

## Results

**Figure 1:**
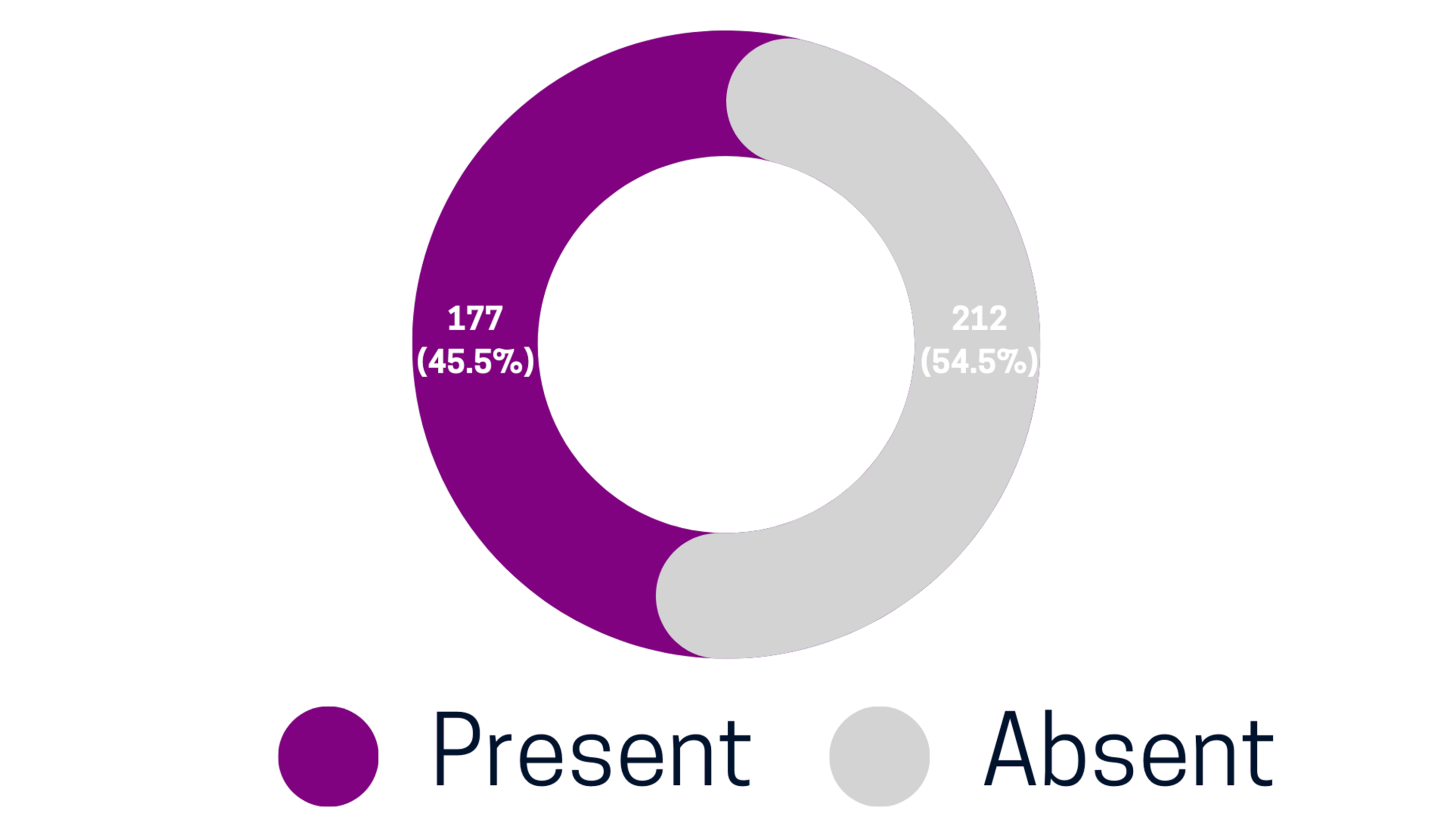
Prevalence of Jawbone Lesions in the Study Population

A total of 389 subjects were included in this study, with a slight female predominance (199 females, 51.2%) compared to males (190 males, 48.8%). The age distribution showed that 122 (31.4%) subjects were between 18 and 31 years old, 162 (41.6%) were between 31 and 45 years old, and 105 (27%) were over 45 years old. Panoramic radiographic analysis identified jawbone lesions in 177 subjects, representing a prevalence of 45.5% (Figure 1).

**Figure 2:**
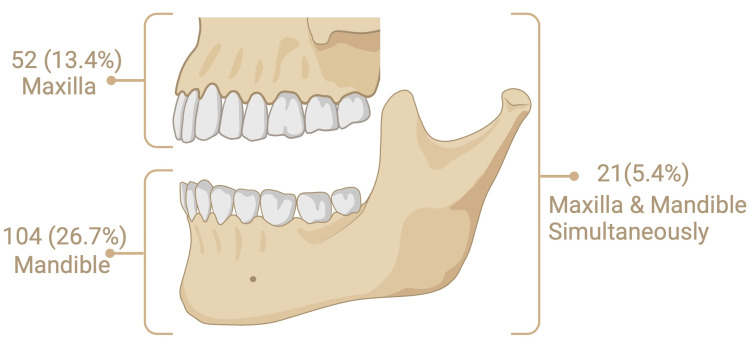
Distribution of jawbone lesions by anatomical location.

Among these, radiolucent lesions were the most common, observed in 153 subjects (39.3% of the total sample and 86.4% of those with lesions), while radiopaque lesions were less frequent, observed in 18 subjects (4.6% of the total sample and 10.2% of those with lesions). A minority of cases (six subjects, 1.5% of the total sample and 3.4% of those with lesions) exhibited both radiolucent and radiopaque lesions, while no cases showed a mixed lesion. The anatomical distribution of these lesions revealed a higher incidence in the mandible, with 104 (26.7%) cases compared to 52 (13.4%) cases in the maxilla. Additionally, 21 (5.4%) cases involved both the maxilla and mandible (Figure 2).

**Figure 3:**
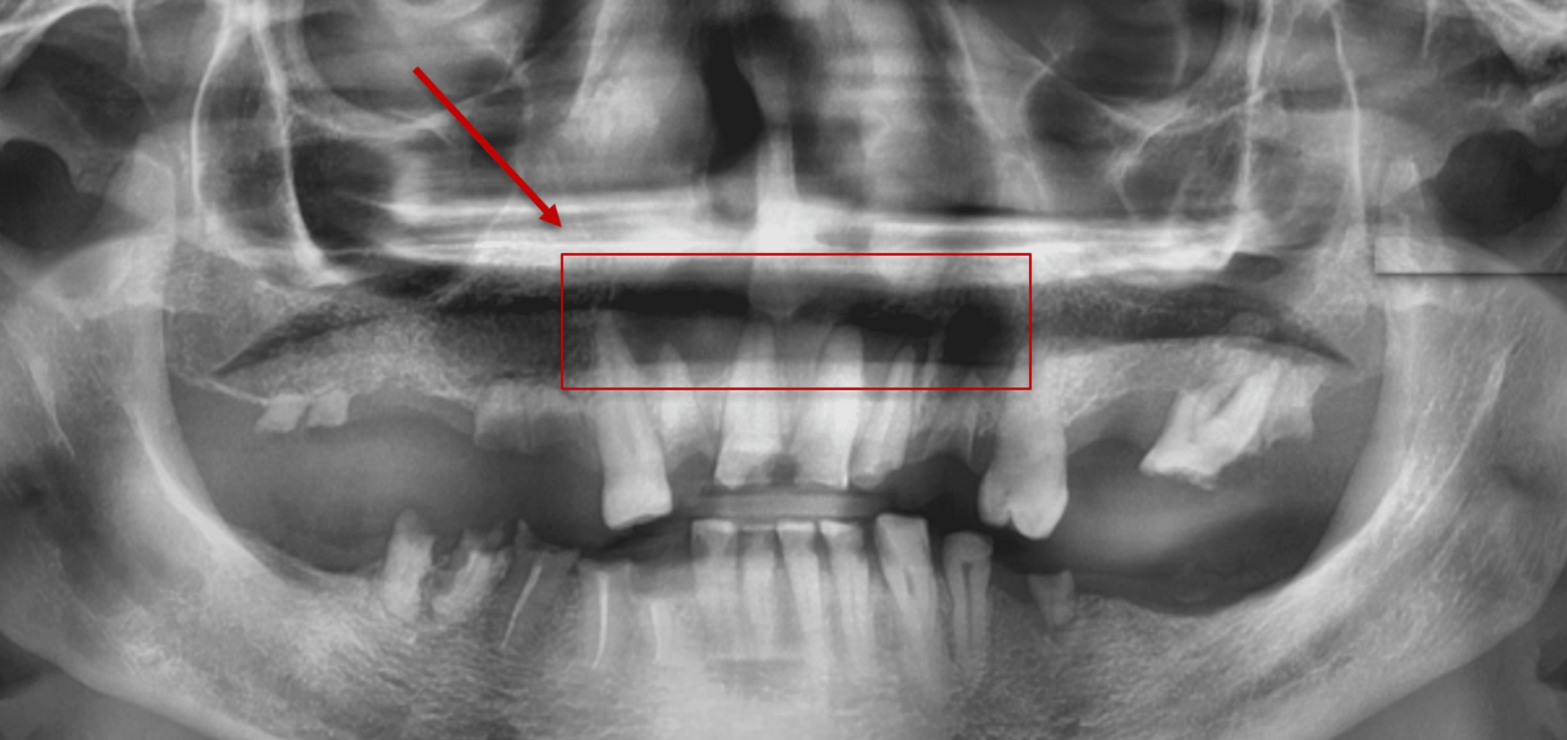
A panoramic radiograph of a 51-year-old male patient showing multiple periapical radiolucent lesions on both sides of the anterior maxillary region.

**Figure 4:**
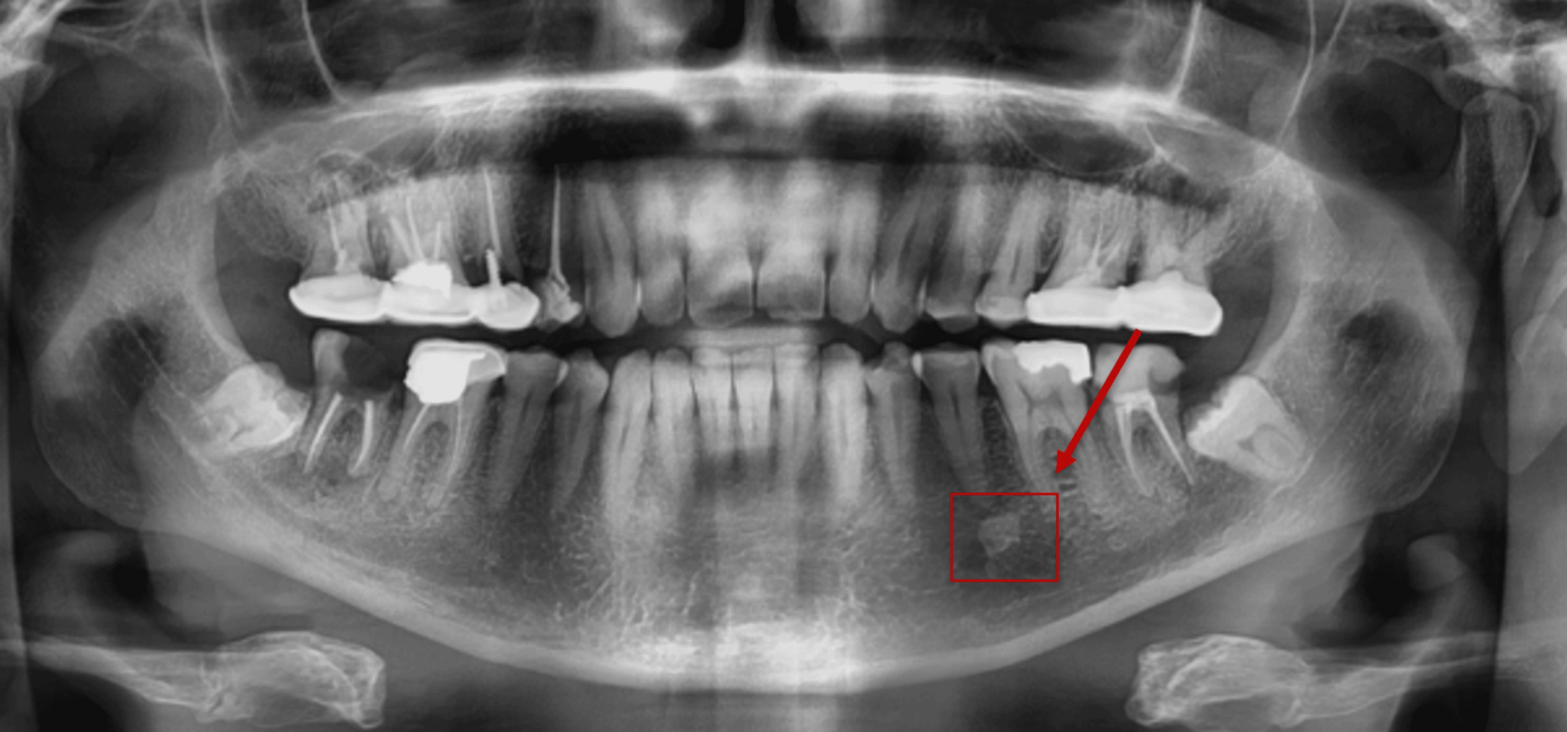
A panoramic radiograph of a 40-year-old male patient showing a periapical radiopaque lesion on the left side of the mandibular posterior region.

**Figure 5:**
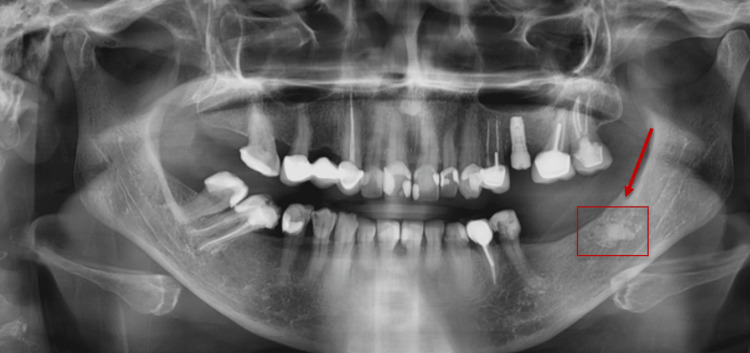
A panoramic radiograph of a 45-year-old female patient showing a radiopaque lesion on the left side of the mandibular edentulous posterior region.

The posterior region of the jaw was most frequently affected, with 124 (31.9%) subjects showing lesions in this area, compared to 33 (8.5%) cases in the anterior region, and 20 (5.1%) cases involving both the anterior and posterior regions. Lesions were equally distributed between the right and left sides of the jaw, each affecting 63 (16.2%) subjects, while 51 (13.1%) subjects involved both sides (Figure 3). Further analysis of the lesion sites revealed that the mandibular posterior teeth were the most commonly affected, with 84 (21.6%) cases, followed by maxillary posterior teeth in 20 (5.1%) cases (Figure 4). Lesions associated with the mandibular anterior teeth were observed in nine (2.3%) cases and maxillary anterior teeth in 14 (3.6%) cases. A significant number of cases (26 subjects, 6.7%) involved multiple sites, while four (1%) cases were edentulous (Figure 5), and 20 (5.1%) cases were identified as isolated jawbone lesions (Table 1).

**Table 1:** Demographic characteristics and distribution of jawbone lesions among the study subjects.

Variables	N (%)
Age group (years)	
18–31	122 (31.4%)
31–45	162 (41.6%)
>45	105 (27%)
Gender	
Male	190 (48.8%)
Female	199 (51.2 %)
Type of lesion	
Radiolucent	153 (39.3%)
Radiopaque	18 (4.6%)
Both	6 (1.5%)
Mixed	0 (0%)
Involvement site	
Anterior	33 (8.5%)
Posterior	124 (31.9%)
Both	20 (5.1%)
Involvement side	
Right	63 (16.2%)
Left	63 (16.2%)
Both	51 (13.1%)
Lesion association	
Mandibular posterior teeth	84 (21.6%)
Maxillary posterior teeth	20 (5.1%)
Mandibular anterior teeth	9 (2.3%)
Maxillary anterior teeth	14 (3.6%)
More than one site	26 (6.7%)
Edentulous	4 (1%)
Isolated jawbone lesion	20 (5.1%)

This study meticulously examined the prevalence and types of jawbone lesions, focusing on various demographic and anatomical factors, to better understand the distribution and characteristics of these lesions in a diverse population. The investigation revealed that gender did not have a significant impact on the presence of jawbone lesions. Specifically, 48.6% of males and 51.4% of females were found to have jawbone lesions, with no statistically significant difference between the genders (p > 0.05). Furthermore, the types of lesions, whether radiolucent, radiopaque, or a combination of both, were similarly distributed between males and females, again showing no significant difference (p > 0.05). This indicates that gender is not a determining factor in either the likelihood of developing jawbone lesions or in the type of lesion that might develop. Regarding age, the study revealed that the prevalence of jawbone lesions varied across different age groups, though not to a statistically significant extent (p > 0.05). Lesions were most commonly observed in the 31-45-year age group, where 42% of participants were affected. The younger age group of 18-30 years had a 35% prevalence, while the older group, those over 45 years, showed a 23% prevalence. This trend suggests that middle-aged individuals might be at a higher risk of developing jawbone lesions, although the lack of statistical significance indicates that age alone may not be a decisive factor. Interestingly, the distribution of lesion types approached statistical significance (p > 0.05), with radiolucent lesions being more common in the younger and middle-aged groups, whereas radiopaque lesions were relatively rare across all age groups. This pattern may reflect age-related changes in bone density or the natural history and progression of jawbone lesions, with younger individuals potentially experiencing different etiologies or stages of lesion development compared to older adults. The anatomical location of the jawbone lesions showed a significant relationship with both their prevalence and type. Lesions were more frequently found in the mandible, with 59% of cases, compared to 29% in the maxilla, and this difference was statistically significant (p < 0.05). Additionally, the type of lesion was significantly associated with the jaw involved, with radiolucent lesions being predominant in the mandible. The maxilla, on the other hand, had a lower incidence of both radiolucent and radiopaque lesions. The remaining 12% of lesions involved both the maxilla and mandible, with these cases often presenting more complex pathology. The significant difference between the jaws may be attributed to anatomical and functional differences. The mandible’s greater involvement in masticatory function and its denser bone structure could contribute to a higher susceptibility to certain types of lesions. There was a significant association between the site of lesion involvement and both the prevalence and type of lesion. Posterior sites of the jaw were affected in 70% of cases, compared to 19% in anterior sites, and this difference was statistically significant (p < 0.05). Lesions in the posterior region were predominantly radiolucent, which may be linked to the higher functional load and greater incidence of inflammatory or degenerative conditions in this area. The anterior region, although less frequently involved, also showed a significant pattern, with fewer lesions and a predominance of radiolucent types. The difference in lesion prevalence and type between anterior and posterior sites underscores the potential impact of localized mechanical forces, as well as the distinct anatomical and physiological characteristics of these regions. Furthermore, the side of the jaw involved in lesions also demonstrated significant differences. Lesions were equally prevalent on the right and left sides of the jaw, with each side being affected in 35.6% of cases. However, 28.8% of cases involved both sides of the jaw, indicating a more extensive or systemic process. The distribution of lesion types also showed a significant association with the side involved, with radiolucent lesions being the most common across all sides. This symmetry in the distribution of lesions might suggest that bilateral factors, such as systemic conditions or generalized bone pathology, play a role in the development of these lesions. Alternatively, the similar involvement of both sides could reflect a balance in mechanical loading and other functional aspects of the jaw that predispose both sides equally to lesion development (Table 2).

**Table 2:** Prevalence and type of jawbone lesions by gender and age.

Variables		Jawbone lesion		Type of lesion			
		Present	P-value	Radiolucent	Radiopaque	Both	P-value
Gender	Male	86 (48.6%)	0.972	73 (41.24%)	9 (5.08%)	4 (2.26%)	0.836
	Female	91 (51.4%)		80 (45.2%)	9 (5.08%)	2 (1.13%)	
Age (years)	18–30	61 (35%)	0.244	53 (29.94%)	4 (2.26%)	4 (2.26%)	0.051
	31–45	75 (42%)		63 (35.59%)	12 (6.78%)	0 (0.0%)	
	>45	41 (23%)		37 (20.9%)	2 (1.13%)	2 (1.13%)	
Jaw involved	Maxilla	52 (29%)	<0.001*	49 (27.68%)	3 (1.69%)	0 (0.0%)	<0.001*
	Mandible	104 (59%)		88 (49.72%)	15 (8.47%)	1 (0.56%)	
	Both	21 (12%)		16 (9.04%)	0 (0.0%)	5 (2.82%)	
Site involved	Anterior	33 (19%)	<0.001*	31 (17.51%)	2 (1.13%)	0 (0.0%)	<0.001*
	Posterior	124 (70%)		105 (59.32%)	16 (9.04%)	3 (1.69%)	
	Both	20 (11%)		17 (9.6%)	0 (0.0%)	3 (1.69%)	
Side involved	Right	63 (35.6%)	<0.001*	50 (28.25%)	12 (6.78%)	1 (0.56%)	<0.001*
	Left	63 (35.6%)		57 (32.2%)	5 (2.82%)	1 (0.56%)	
	Both	51 (28.8%)		46 (25.99%)	1 (0.56%)	4 (2.26%)	

This study thoroughly examined the distribution of jawbone lesions concerning gender, age, and the anatomical side involved, revealing nuanced relationships between these variables and the occurrence of lesions in various jaw regions. Gender did not significantly influence the distribution of jawbone lesions (p > 0.05), with males showing a higher prevalence in the mandibular posterior teeth region (21.9%) and lower occurrences in the maxillary posterior teeth (6.2%), mandibular anterior teeth (2.3%), and maxillary anterior teeth (3.4%). Males also had 9.6% of lesions in more than one site, 0.6% were edentulous, and 4.5% had isolated lesions. Similarly, females exhibited lesions primarily in the mandibular posterior teeth (25.4%), followed by the maxillary posterior teeth (5.1%), mandibular anterior teeth (2.8%), and maxillary anterior teeth (4.5%), with 5.1% having lesions in more than one site, 1.7% being edentulous, and 6.8% having isolated lesions. Although females had a slightly higher prevalence in most regions, these differences were not statistically significant. Age showed a significant association with the distribution of jawbone lesions (p < 0.05), with the highest prevalence in the 31-45-year age group (24.3% in the mandibular posterior teeth), followed by the 18-30-year group (17.5%) and the >45-year group (5.6%). The youngest age group showed 7.3% of lesions in more than one site, while the oldest group had a relatively higher prevalence of 4.5% in multiple sites, suggesting that middle-aged adults are more susceptible to jawbone lesions, particularly in the mandibular regions, possibly due to cumulative dental stress, changes in bone density, or increased exposure to risk factors. The side of the jaw involved also showed a significant association with lesion distribution (p < 0.05), with a higher prevalence on the right side, where 22.6% had lesions in the mandibular posterior teeth, and a slightly lower prevalence on the left side (19.2%). Both sides were affected in 5.6% of cases, often with more complex lesion patterns, including 10.7% involving multiple sites. The significant differences between the sides may be influenced by functional load during mastication, handedness, or anatomical variations (Table 3).

**Table 3:** Distribution area of jawbone lesions by gender, age, and side involvement.

Variables		Associated area							P-value
		Mandibular posterior teeth	Maxillary posterior teeth	Mandibular anterior teeth	Maxillary anterior teeth	More than one site	Edentulous	Isolated jawbone lesion	
Gender	Male	39 (21.9%)	11 (6.2%)	4 (2.3%)	6 (3.4%)	17 (9.6%)	1 (0.6%)	8 (4.5%)	0.641
	Female	45 (25.4%)	9 (5.1%)	5 (2.8%)	8 (4.5%)	9 (5.1%)	3 (1.7%)	12 (6.8%)	
Age (years)	18–30	31 (17.5%)	5 (2.8%)	3 (1.7%)	3 (1.7%)	13 (7.3%)	1 (0.6%)	5 (2.8%)	0.016*
	31–45	43 (24.3%)	6 (3.4%)	6 (3.4%)	5 (2.8%)	5 (2.8%)	1 (0.6%)	9 (5.1%)	
	>45	10 (5.6%)	9 (5.1%)	0 (0%)	6 (3.4%)	8 (4.5%)	2 (1.1%)	6 (3.4%)	
Side involved	Right	40 (22.6%)	8 (4.5%)	2 (1.1%)	5 (2.8%)	4 (2.3%)	0 (0%)	4 (2.3%)	<0.001*
	Left	34 (19.2%)	6 (3.4%)	5 (2.8%)	2 (1.1%)	3 (1.7%)	2 (1.1%)	11 (6.2%)	
	Both	10 (5.6%)	6 (3.4%)	2 (1.1%)	7 (4.0%)	19 (10.7%)	2 (1.1%)	5 (2.8%)	

## Discussion

In this retrospective, cross-sectional study, 177 out of 389 participants from Hail, Saudi Arabia, were found to have jawbone lesions, representing a 45.5% prevalence. The gender distribution within the study sample demonstrated a nearly equal representation, with males comprising 48.8% and females 51.2% of the participants. This balanced distribution facilitates an unbiased comparison between genders, addressing and potentially refuting pre-existing assumptions regarding gender-based predisposition to jawbone lesions.

The prevalence of jawbone lesions observed in our study, at 45.5%, is notably higher than the 30.62% prevalence reported by Dantu and Puranik (2017) in the Bagalkot population. This significant difference may be attributed to a variety of factors, including genetic predisposition, environmental exposure, and the difference in sample sizes between the studies. Our study involved 389 participants, whereas Dantu and Puranik included a much larger sample size of more than 3,500 participants [10].

In this study, radiolucent lesions were the most prevalent jawbone lesions, observed in 153 subjects representing 39.3% of the total sample. Radiopaque lesions were observed in 18 subjects, representing 4.6%, while a minor subset of six (1.5%) subjects exhibited both radiolucent and radiopaque features, with no cases showing mixed lesions. Another study conducted by the University of Florida, which focused on the prevalence of radiolucent and radiopaque lesions, found that radiolucent lesions were more common than radiopaque lesions, which is consistent with our findings [11]. However, the distribution and prevalence rates varied due to the specific population studied, environmental factors, and diagnostic methodologies used. The consistently higher prevalence of radiolucent lesions observed in this study highlights the significant incidence of conditions such as cysts and inflammatory lesions across various populations. This finding suggests that these pathologies are more commonly encountered in clinical practice, necessitating careful consideration during diagnosis and treatment planning. In contrast, the lower prevalence of radiopaque lesions points to their association with bone-forming processes or sclerotic changes, which occur less frequently. Given the predominance of radiolucent lesions, this study underscores the critical importance of focusing on their identification and management within both medical and dental fields, as they represent the most common type of jawbone lesions in diverse demographic groups.

A study published in 2021 reported the prevalence of jawbone lesions in a specific population group, focusing on age-related trends. The study found that the prevalence of jawbone lesions, both radiolucent and radiopaque, increased with age. The highest prevalence was observed among participants aged 60 years or older, suggesting that age is an important factor in the development of these lesions. This increase in prevalence with age can be attributed to age-related changes in bone density, prolonged exposure to risk factors, and the cumulative effects of chronic conditions that affect oral health [12]. However, the prevalence of jawbone lesions exhibited a 15.86% in the 18-30-year age group, 19.29%in the 31-45-year age group, and 10.54% in individuals over 45 years of age, according to the age-related analysis conducted in this study. Although the decreased percentage of lesions was observed with an increase in age, statistical analysis revealed no significant difference (p > 0.05), which may be due to the number of included samples that show the lowest percentage of those older than 45 years.

The anatomical distribution of these lesions enriches our understanding of their nature. The mandible, which has a strong anatomical structure, was the most affected by lesions, with 104 (59%) cases. The maxilla, although less commonly affected, was affected in 52 (29%) cases. In addition, 21 (12%) cases showed lesions in both the maxilla and mandible, and these findings were statistically significant (p < 0.05). This distribution suggests a possible difference in susceptibility between the mandible and maxilla, which may be influenced by factors such as vascularization, mechanical stress, or developmental differences. The results of our study are in complete agreement with those of another study conducted in 2008 in northern Greece, which suggested that the cause of the localization of lesions in the mandible is differences in bone anatomy and blood supply or mechanical load, or developmental origins that make the mandible more susceptible to bearing a higher lesion burden [13].

This study identified that the mandibular posterior teeth region was more susceptible to lesions (21.6%) compared to other regions within the jaws. These findings confirm earlier research by Kawai et al. (1992), which showed that lesions predominantly localized in the mandible, and the most frequently affected site was the mandibular first molar region, accounting for approximately 36% of all lesions, followed by the mandibular second molar region, which comprised around 26% of the cases [14].

The analysis of jawbone lesions revealed a nearly symmetrical distribution between the right and left sides of the jaw. Specifically, 35.6% of the study population exhibited right-sided lesions, an identical 35.6% presented with left-sided lesions, and 28.8% had lesions involving both sides of the jaw, with statistical significance (p < 0.05). Corroborating these findings, a study by Ba-Hattab et al. similarly examined the laterality of jawbone lesions, reporting an equivalent distribution across both sides [15].

Clinically, these findings suggest that special attention should be given to the mandible, particularly the posterior region, in both diagnostic and treatment planning due to its higher susceptibility to lesions. The significant association between side involvement and lesion prevalence highlights the need for thorough bilateral examination in patients, especially when symptoms are unilateral. Future studies should explore the biological mechanisms contributing to these patterns, particularly concerning age and lesion distribution, to develop targeted interventions that could mitigate the risk of jawbone lesions.

Limitations

This study has several limitations. The relatively small sample size of 389 panoramic radiographs and the focus on a specific population in Hail, Saudi Arabia, may limit the generalizability of the findings. Variations in image quality and the potential for observer bias also affect the accuracy of lesion detection and classification. Additionally, the study did not correlate radiographic findings with clinical or histopathological data, limiting the ability to confirm the nature of the lesions.

## Conclusions

This study identified the prevalence of jawbone lesions in the population of Hail, Saudi Arabia, using panoramic radiographic analysis. The findings revealed a significant prevalence of jawbone lesions, with nearly half of the studied population affected (45.5%). Radiolucent lesions were the most common type, predominantly affecting the posterior region of the mandible. These insights underline the importance of targeted dental health initiatives and early diagnostic measures. The study’s thorough radiographic analysis provides a critical understanding of the distribution and characteristics of jawbone lesions in this region, serving as a valuable reference for future research and public health strategies.
